# Novel Technologies to Address the Lower Motor Neuron Injury and Augment Reconstruction in Spinal Cord Injury

**DOI:** 10.3390/cells13141231

**Published:** 2024-07-22

**Authors:** Stanley F. Bazarek, Matthias J. Krenn, Sameer B. Shah, Ross M. Mandeville, Justin M. Brown

**Affiliations:** 1 Department of Neurosurgery, Massachusetts General Hospital, Harvard Medical School, Boston, MA 02114, USA; sbazarek@bwh.harvard.edu (S.F.B.); mkrenn@mgh.harvard.edu (M.J.K.); rmmandeville@mgb.org (R.M.M.); 2 Department of Neurological Surgery, University Hospitals-Cleveland Medical Center, Case Western Reserve University School of Medicine, Cleveland, OH 44106, USA; 3 Department of Neurosurgery, University of Mississippi Medical Center, Jackson, MS 39216, USA; 4 Center for Neuroscience and Neurological Recovery, Methodist Rehabilitation Center, Jackson, MS 39216, USA; 5 Spinal Cord Injury Medicine and Research Services, VA Medical Center, Jackson, MS 39216, USA; 6 Departments of Orthopedic Surgery and Bioengineering, University of California-San Diego, La Jolla, CA 92093, USA; sbshah@ucsd.edu; 7 Research Division, VA San Diego Medical Center, San Diego, CA 92161, USA

**Keywords:** denervation atrophy, nerve transfer, electrical stimulation, cell transplantation, cauda equina, conus medullaris, stem cell therapy

## Abstract

Lower motor neuron (LMN) damage results in denervation of the associated muscle targets and is a significant yet under-appreciated component of spinal cord injury (SCI). Denervated muscle undergoes a progressive degeneration and fibro-fatty infiltration that eventually renders the muscle non-viable unless reinnervated within a limited time window. The distal nerve deprived of axons also undergoes degeneration and fibrosis making it less receptive to axons. In this review, we describe the LMN injury associated with SCI and its clinical consequences. The process of degeneration of the muscle and nerve is broken down into the primary components of the neuromuscular circuit and reviewed, including the nerve and Schwann cells, the neuromuscular junction, and the muscle. Finally, we discuss three promising strategies to reverse denervation atrophy. These include providing surrogate axons from local sources; introducing stem cell-derived spinal motor neurons into the nerve to provide the missing axons; and finally, instituting a training program of high-energy electrical stimulation to directly rehabilitate these muscles. Successful interventions for denervation atrophy would significantly expand reconstructive options for cervical SCI and could be transformative for the predominantly LMN injuries of the conus medullaris and cauda equina.

## 1. Introduction

Following injury, the central nervous system (CNS) prioritizes preservation of vital tissue over regeneration. Therefore, glial scarring is the rule and plasticity of the remaining connections is the primary strategy for functional recovery, rather than axon regeneration [1,2]. This restrictive environment of the CNS has led to clinical efforts focused on bypassing the central lesion via manipulations of the peripheral nervous system (PNS) to achieve specific functional goals. Such interventions include methods such as brain–computer interfaces (BCI) [3], tendon transfers [4,5], and nerve transfers [6]. Tendon and nerve transfers redistribute preserved, volitional movements to more essential targets to maximize overall function [6]. Such transfers require adequate remaining control and therefore, few options exist above a C5 level of spinal cord injury (SCI). Rostral to this, BCI becomes an important consideration given the limited volitional movements to redistribute. BCIs interpret signals directly from the brain [3,7], which are then relayed to peripheral targets via functional electrical stimulation (FES) for execution of the desired function [8,9]. 

In contrast to the CNS, the PNS has the capacity to regenerate, albeit slowly. Axonal growth occurs at approximately 1 mm per day [10]. A major obstacle to this axon regeneration is denervation atrophy, a progressive and eventually irreversible degeneration of its target muscle with fatty and fibrotic infiltration of the muscle [11]. Similar changes also occur in the distal nerve sheaths which are deprived of axons [12]. This degenerative process places a time constraint on nerve transfer options which should be performed within a year, but ideally before 6 months from the time of injury [13]. Long-distance targets, such as reinnervating the extent of a limb (i.e., proximal ulnar nerve injury to the intrinsic muscles of the hand), are considered unlikely to succeed even with immediate repair due to irreversible degeneration taking place by the time the slowly regenerating axons reach the target. 

**Figure 1:**
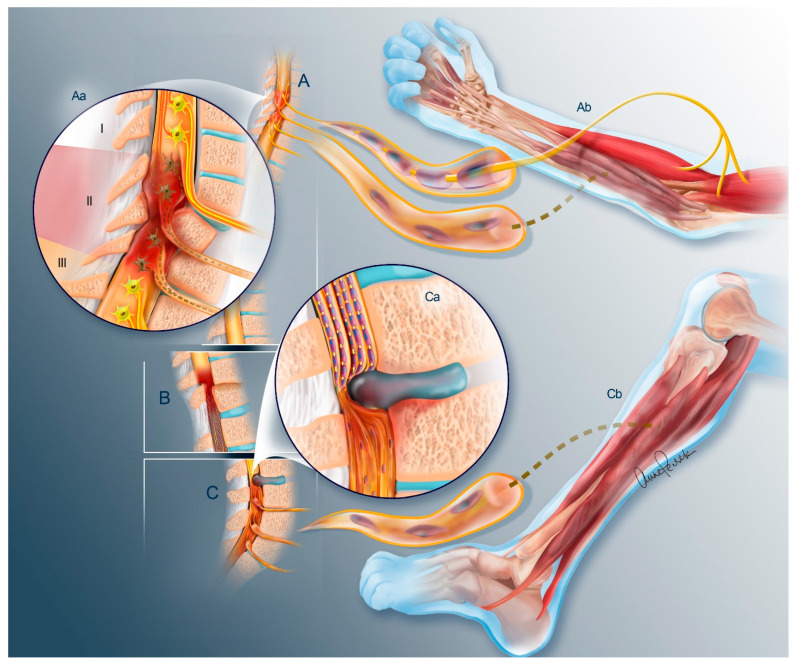
Lower Motor Neuron Spinal Cord Injury. (**A**) Cervical SCI results in (Aa) three regions of the spinal cord: (I) the supralesional segment rostral to the injury site that remains healthy and innervates the preserved muscle groups (superior nerve in (Ab)); (II) the injured metamere where tissue damage to the spinal cord disrupts the SMNs and their associated peripheral axons ((Ab) lower nerve); and (III) the infralesional segment which has lost its descending white matter connections, but whose SMNs are preserved with their associated peripheral axons. The axons of the SMNs comprise the motor axons of the PNS and their loss results in denervation and subsequent degeneration of the target muscle (Ab). (**B**) The conus medullaris is the last segment of the spinal cord proper and is the location of the SMNs that innervate the lower extremities as well as bowel, bladder, and sexual functions. (**C**) Only nerve roots exist caudal to the conus, known as the cauda equina (3a). Injuries at this level sever the axons of the SMNs (Ca) resulting in denervation of the same targets. Injuries to the conus and cauda equina are lower motor injuries and must be addressed before irreversible neuromuscular degeneration is established (Cb). Healthy SMN cell bodies and axons are in yellow and degenerated SMNs are brown with dotted or absent axons. Descending, healthy UMN axons in (Aa) are shown in orange.

**Figure 2:**
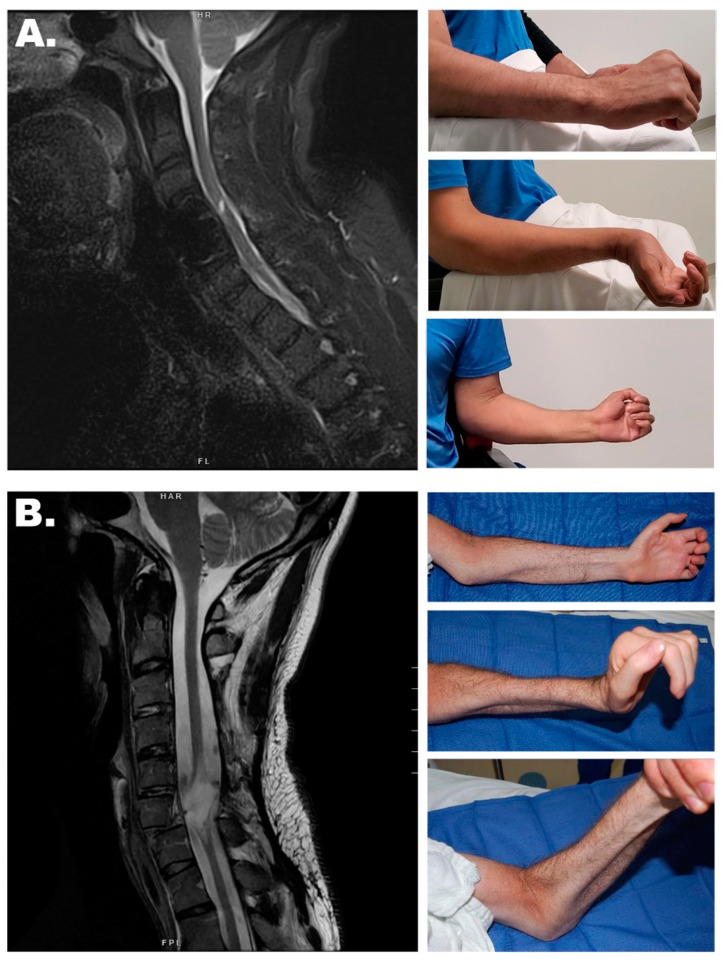
Signs of Denervation Atrophy. (**A**) This cervical MRI demonstrates a limited spinal cord injury. In spite of the fact that this was a C6 motor complete SCI, there is very little loss of gray matter and the paralyzed muscles of the forearm and hand remain innervated and therefore maintain substantial muscle bulk and tone. (**B**) In this C7 motor complete SCI, the cervical MRI shows extensive destruction of spinal cord tissue. This tissue contains the SMNs and their destruction results in Wallerian degeneration of their associated peripheral axons. This LMN injury results in severe atrophy of the associated muscles in the forearm and hand.

While less commonly discussed as a contribution to paralysis due to SCI, lower motor neuron (LMN) injury is often an important contributor to disability (Figure 1) [14]. This is clear in conus and cauda equina injuries where all or at least the vast majority of the disability is due to LMN injury [15]. Similarly, cervical SCI, in which arm and hand function is impaired, may also have an important LMN contribution due to direct injury to the spinal motor neurons (SMNs) within the gray matter at the site of impact. This can result in notable atrophy of the forearms and hands (Figure 2). When the spinal cord is injured, both the peripheral white matter and the central gray matter are injured at the site of impact. This can involve a discrete region of the spinal cord (Figure 2A) or it can span many segments (Figure 2B). Therefore, a mixed injury pattern occurs that involves disruption of both descending upper motor neuron (UMN) fibers from the brain/brainstem, as well as the SMNs whose axons exit the cord as peripheral nerves to innervate the muscles of the upper extremity (Figure 1A). As described above, injury to these SMNs will result in denervation atrophy of these targets and eventual loss of their potential to be reanimated through either BCI/FES or nerve transfer strategies. Frequently, referrals for surgical reanimation are not made within the allotted time window and reconstructive options are therefore no longer available to these patients.

In order to expand this limited time window for intervention, several strategies have been proposed. The following methods will be discussed in this review: the “babysitter” nerve transfer, stem cell-derived SMN transplantation, and high-energy electrical muscle stimulation (EMS). Successful prevention of denervation atrophy would be transformative for functional recovery. This could allow for a number of additional nerve transfer-type interventions, even expanding into the lower extremities. Furthermore, reinnervation would offer the potential for FES strategies [16]. 

## 2. The Problem of Denervation Atrophy

**Figure 3:**
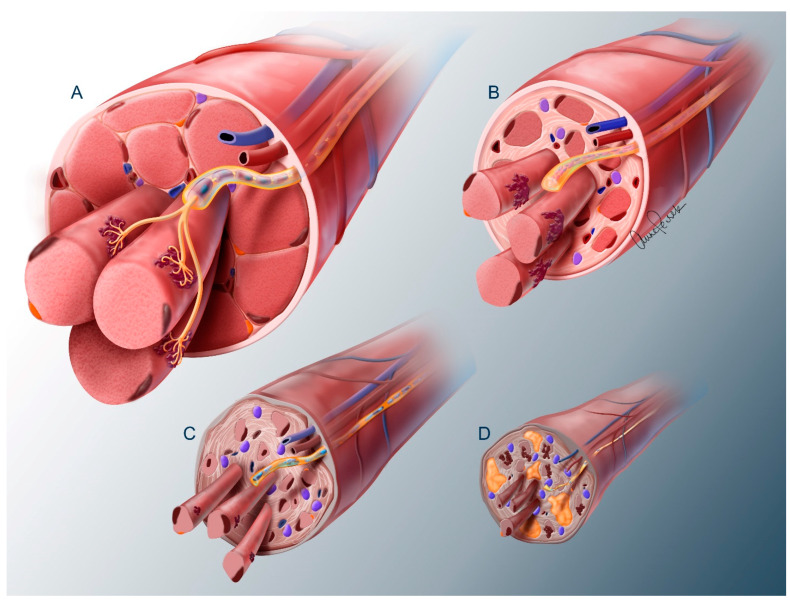
Neuromuscular Degeneration following Denervation. View of a terminal nerve entering a muscle fascicle. (**A**) A normal, uninjured nerve associated with large muscle fibers and minimal endomysial connective tissue. Associated satellite cells are seen (orange cells) beneath the membrane of the muscle fiber. The axon exits the terminal intramuscular sheath to form a complex NMJ on three fibers. (**B**) At 8 months post-denervation, there is significant muscle fiber atrophy. There is proliferation and differentiation of fibro-adipogenic progenitors (FAPs) (purple cells) with increased endomysial fibrosis. Schwann cells (SC) within the terminal sheaths have transitioned to a regenerative phenotype forming Bands of Büngner, but these SC numbers have now begun to decline. Good recovery may still be achieved at this point if innervation is restored. (**C**) At 2 years post-denervation, there is further atrophy of the muscle fibers with breakdown of the contractile apparatus (punctate appearance in the healthy muscle). The SCs lose their regenerative phenotype with a corresponding decline in production of neurotrophins and fibrosis within the nerve sheath progresses. The NMJ shows fragmentation and a more plaque-like morphology. (**D**) At 5 years post-denervation, there is severe fiber atrophy and extensive fatty (yellow globules) and fibrotic infiltration, including the intramuscular nerve sheath and associated microvasculature. Some fibers exhibit nuclear clumping interspersed with empty appearing cytoplasm. NMJs may persist but are dysmorphic with a small plaque-like appearance.

In our experience, nerve transfers are generally successful many years after injury if the recipient muscle remains innervated by SMNs which originate below the site of trauma and are therefore preserved (Figure 1Aa Region III and Figure 2A) [17]. These muscles will often exhibit increased tone and neuromuscular electrical stimulation (NMES) can be used to confirm this preserved innervation by activating the paralyzed muscles with a full contraction [18]. When the recipient muscle is not innervated and therefore does not respond to NMES, a nerve transfer should be performed early to avoid the progressive degeneration that inevitably ensues in the absence of these axons (Figure 2B). This response of the neuromuscular circuit to axon deprivation has been well described (Figure 3) [10]. In fact, a number of barriers to axon reintroduction have been identified within both the nerve and muscle. These barriers will be discussed below.

### 2.1. Nerve Trunk

In SCI, the peripheral axons associated with the SMNs which are lost or destroyed will undergo Wallerian degeneration (Figure 1Ab) [19]. This involves dissolution of the axon and its myelin sheath. As the myelin sheaths are lysed, the associated Schwann cells (SCs) transition to a repair phenotype [20]. They proliferate in this process to prepare to facilitate reinnervation. This process involves the clearance of myelin debris, the formation of axon guidance tracks, and the secretion of pro-regenerative trophic factors. These repair cells break down their own myelin in addition to recruiting macrophages to phagocytose debris. They elongate (up to three-fold), branch, and align themselves to assemble regeneration tracks termed Büngner bands. However, this supportive environment is limited. If axons are not reintroduced via nerve transfer surgery in a timely fashion, the elevated SC numbers will eventually regress with loss of both the repair phenotype and the associated reduction in the release of neurotrophic factors. This is eventually followed by scar formation within endoneurial tubes that remain vacant [21].

With chronic denervation due to loss of SMNs, the associated peripheral nerve itself becomes less favorable for axon regeneration. Less than half of motor axons are shown to traverse a graft which has been without axons for 3 months, compared to a graft that was applied with no delay [12,22]. This initial decline then slows down and reaches a plateau at 6 months. There never appears to be a complete inability to convey axons through the graft even at much greater periods of axon deprivation [12,21,23]. In fact, a nerve graft denervated up to 500 days, while conveying fewer axons, is still able to achieve a tetanic force near that of acutely prepared grafts in a rodent sciatic nerve repair model when coapted to a newly transected distal target. The maintenance of force despite lost axons is due to collateral innervation and the formation of large motor units, which may compensate for up to 80% of axon loss [24].

SC depletion has been suggested as a major contributor to this deficit in axon regeneration. However, the most dramatic decline in axon regeneration is seen at 3 months of denervation in rodents, before there is any significant loss in SC number. In keeping with this finding, nerve regeneration was unchanged in an experimental mouse model that prevented SC proliferation [20]. It has been proposed that it is not the loss of these cells, but the loss of the repair phenotype and associated Büngner bands with depletion of neurotrophic factors that is responsible for the reduction in axon conveyance [20]. In fact, forced expression of the transcription factor c-Jun in SCs, which has been shown to correlate with the repair phenotype, was shown to increase axon regeneration across a chronically denervated nerve segment [25,26]. Contact with a regenerating axon has been shown to induce regenerative genes in SCs following even 8 months of denervation in rats [27]. SCs isolated from chronically denervated nerve segments show similar in vitro properties compared to those isolated from non-injured nerves [21,28]. These findings suggest that SCs remain plastic and amenable to molecular intervention. 

Another impediment to axon receptivity appears to be a progressive fibrosis within the axon-deprived endoneurial tube. This fibrosis, however, may be more a consequence of chondroitin sulphate proteoglycan accumulation, rather than collagen deposition [21]. While collagen is a primary physical barrier to axon growth, effectively narrowing the cross-sectional area through which axons may traverse, chondroitin may be less restrictive and more permissive to regenerating axons. In keeping with this, the enzyme chondroitinase has been shown to promote axon regeneration through glial scar tissue in both PNI [29] and SCI [30]. 

### 2.2. Neuromuscular Junction

Following denervation in mice, pre-existing motor end plates of the neuromuscular junction (NMJ) progressively fragment, disperse, and eventually disappear after a few months [31]. These motor end plate changes have been proposed as a primary impediment to recovery following chronic denervation [32,33]. However, while human NMJs do show some fragmentation over time and a progression from a pretzel-like to a more plaque-like morphology, these fragments can persist for at least 3 years post-denervation [34]. Regardless, it remains unclear whether this change in morphology affects their receptivity to motor axons. There is also evidence that NMJs may form de novo, as demonstrated by insertion of a transected nerve trunk into denervated muscle (direct muscle neurotization) [35]. A more recent report demonstrates that human synapses have a very distinct proteomic profile and remain stable with aging, in comparison to rodents [36]. The significance of the persistence and molecular differences between rodent and human NMJs is unclear and underscores the need for further human studies and caution in the translation of previous rodent reports. 

### 2.3. Muscle

The timeline for denervation atrophy has been characterized in rat muscle [11]. It was found that within 2 months, muscle undergoes rapid atrophy, but remains capable of full recovery with reinnervation. Following this, there is further atrophy and a gradual degeneration of the contractile apparatus, resulting in a progressive decline in restorative capacity. By 7 months, there is little to no recovery with severely atrophied muscle fibers embedded in fatty and fibrotic tissue and drastic involution of the local microvasculature [37,38]. 

This process is similar but more prolonged in humans [39]. Carraro and colleagues investigated these changes in humans through muscle biopsies in patients following lower extremity denervation from conus medullaris or cauda equina injuries [40,41]. Humans undergo muscle atrophy within weeks following denervation, which declines to 10–20% of normal muscle size by 2–3 years [42]. There is a progressive loss of myonuclei and the myofibrillar structure. An increasing subset of fibers show a unique pattern of centralized nuclear clumping interspersed by gaps of empty myoplasm. This may be the final stage of fiber atrophy and the proportion of these fibers decreases after 6 years of denervation in humans where they are predominantly replaced by fat and fibrous tissue [40]. 

Satellite cells are the muscle progenitor cells that reside between the basal lamina and plasma membrane of the muscle fiber. They can be identified by their expression of transcription factor, Pax7 [43,44]. Following denervation in rodents, satellite cells proliferate and generate new muscle fibers that peak at 2 to 4 months, then ultimately succumb to denervation atrophy as do the native fibers. After this initial period, there is a persistence of myogenesis seen at very low levels (1–2% of fibers) within the basal lamina of previous fibers that is observed even after two decades in humans [41]. These new fibers remain very small with central nuclei and do not achieve full differentiation. They stain for the antibody to the embryonic isoform of the heavy myosin chain [42]. The prevailing consensus is that the satellite reserve is elevated and then depleted over time. However, a more recent report using the Pax7 marker claims that the satellite cell population does not significantly decline and is equivalent to uninjured controls at 1 year in rodents [43]. This group also demonstrated that satellite cells harvested from chronically denervated animals show equivalent proliferative and muscle regenerating capacity when transplanted into uninjured animals. A regenerative response is also demonstrated in chronically denervated muscle after direct tissue injury [43,45]. Thus, some potential for regeneration appears to persist chronically.

Recently, the fibro-adipogenic progenitor cell (FAP) was identified within the extracellular matrix of muscle [46,47,48]. Following acute muscle trauma, the FAPs proliferate and differentiate into fibroblasts and deposit collagen to facilitate structural repair, then shortly fall back to normal levels. Following denervation, though, these cells continue to deposit collagen and fat. Further understanding of the mechanisms of the FAP response may provide future drug delivery strategies to mitigate fibrosis, such as targeting the TGFB pathway [49,50]. It has been proposed by several groups that a primary impediment to recovery following chronic denervation is the fibrotic infiltration within the endo- and perimysium of the chronically denervated muscle containing the capillaries and terminal nerve fibers [51,52]. The fibrosis may create a physical barrier within the intramuscular sheaths obstructing axon regeneration through this most distal segment before it can reach the NMJ.

## 3. Strategies to Preserve Neuromuscular Viability Following Denervation

**Figure 4:**
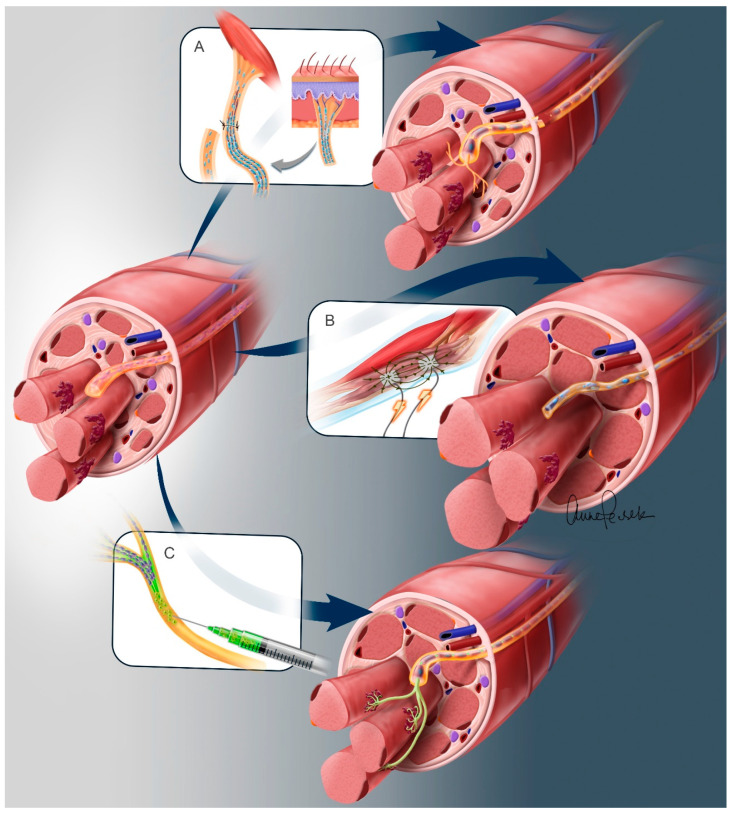
Therapeutic Interventions to Preserve Neuromuscular Integrity Following Denervation Injury. Three interventions are depicted to halt or reverse the degenerative changes associated with denervation. The success of these interventions will likely be time-dependent and should occur before progression to a severe atrophic state. Here we show intervention within a year of denervation. (**A**) **Sensory protection.** A cutaneous nerve is transferred to the target muscle. These axons may preserve the Schwann cells within the nerve and inhibit nerve scarring. The sensory axons cannot form NMJs and thus muscle preservation may be via indirect actions (i.e., neurotrophic factors). (**B**) **Electrical muscle stimulation** can reverse muscle atrophy and may halt fibrotic progression. Although EMS may not reverse the changes within the nerve environment, it may prevent fibrotic infiltration of the intramuscular sheath and potentially allow for delayed innervation. (**C**) **SMN transplantation** offers the possibility to maintain all aspects of the neuromuscular circuit, including the integrity of the nerve, the muscle, and the NMJ. This intervention does not require the sacrifice of a donor motor or sensory nerve.

We will now discuss several therapeutic options that may preserve or even restore neuromuscular integrity for eventual definitive nerve transfer or FES intervention. These include sensory nerve protection, exogenous motor neuron transplantation, and electrical activation of the denervated muscle (Figure 4).

### 3.1. Nerve Transfers 

Following predominantly UMN SCI paralysis where SMNs are predominantly preserved (Figure 2A), nerve transfers have been found to be effective even many years following the injury. Conversely, following SCI with extensive SMN loss (Figure 2B), nerve transfers have been found to only be effective in recovering the denervated target muscles for about a year following such an injury [6]. Within this time frame, these muscles are successfully reinnervated to varying degrees and can achieve reasonable strength and control. Beyond this time point, the efficacy of these procedures appears to wane in proportion to the elapsed time from injury [13].

Nerve transfers redistribute preserved nerves which originate rostral to the site of injury (Figure 1Aa Region I) with redundant function to restore critical functions. Most nerve transfers for restoring hand function in tetraplegia are relatively short-distance transfers and do not require significant time for regeneration. Unfortunately, such nerve transfers have not been applied in the same way to restore leg function following conus and cauda equina injuries. This is due to the significant distances of regeneration that would be required for reinnervation of these targets. The time it would take the transferred axons to reach these targets would generally be longer than the window of receptivity discussed above. Problems like this have led to strategies to halt this degeneration in order to make such interventions more feasible.

One of those strategies is the “babysitter” transfer. A “babysitter” nerve transfer involves the transfer of adjacent motor axons that may not be appropriate to drive function, but sufficient to delay degeneration. This has been applied in the setting of upper extremity nerve injuries (i.e., proximal ulnar nerve) [53] and even facial paralysis when planning reinnervation from the contralateral face [54]. In these scenarios, a nearby nerve is used to occupy the target muscle until the desired axons can make their journey to the target. In the conus/cauda scenario, there are typically no similar options for nearby motor axons that can occupy the target muscle and therefore this strategy is not viable. 

#### Sensory Preservation

While there may be no available motor axon sources following SCI, there are typically abundant sensory axon sources. This is because the dorsal root ganglia (containing the sensory neurons, located outside the spinal cord) and their associated axons are generally preserved in these cases. Several reports have suggested a potential benefit in using a sensory nerve as a babysitter, termed “sensory protection” (See Review [55]). While these sensory axons cannot form NMJs, they offer the potential to occupy the intramuscular sheaths and thereby preserve the neural architecture, preventing endoneurial fibrosis and maintaining the resident SCs [52]. Neurotrophic factors released by the nerve terminal have also been proposed as a method of preserving the target muscle [51,56]. 

Reports have generally shown that sensory protection offers some benefit compared to unprotected controls [51,55,57] but is clearly less robust than that provided by motor axons [58]. One clinical case report demonstrated reanimation of the tibialis anterior and gastrocnemius muscles following an end-to-side transfer of the saphenous nerve into the respective tibial and common peroneal branches following a proximal sciatic nerve injury [59]. Muscles distal to the knee that did not receive this sensory protection failed to show signs of recovery. The sensory axons enter the target nerve through a perineural window cut near the muscle target and may achieve preservation of the distal endoneurial channels while avoiding any competition for NMJs by the regenerating motor axons that would arrive in a more delayed fashion. In another rodent study, isolation of a denervated muscle that had undergone sensory protection within a silicone wrap to prevent any ectopic motor fiber innervation demonstrated no gross benefit to muscle size or architecture. This suggests that some of the studies that have demonstrated an effect may be presenting a false positive due to motor axons having inadvertently arrived at the destination being assessed [60]. It remains unclear as to the extent to which sensory axons might preserve muscle or NMJ integrity in the absence of motor axons.

### 3.2. Spinal Motor Neuron Transplantation 

As practical motor axon donors for nerve transfers are very limited in SCI, an alternative source of axons can be provided via the transplantation of stem-cell-derived SMNs [61]. SMNs can be transplanted into the distal nerve trunk and these cells can both survive and then send axons to innervate and preserve one or more targets. While such cells would not convey any useful function as they are otherwise isolated from the nervous system, these neurons could serve to babysit muscle until long-distance regenerating axons arrive, similar to the motor nerve babysitter transfers described above. Alternatively, they could remain indefinitely for providing muscle bulk and tone, as seen in UMN lesions. In SCI hands, such preservation of paralyzed intrinsic muscles of the hands can be quite useful in maintaining a healthy resting hand posture which allows better dynamics of the hand when the extrinsic muscles are functionally reinnervated by a nerve transfer (Figure 2A). Finally, having such cells in place innervating otherwise paralyzed muscles would provide additional options for FES interventions as in the BCI scenario described in the introduction [62].

Advances in stem cell biology have enabled the generation of human pluripotent stem cells [63] and their subsequent differentiation to specific cell types, including neuronal subtypes [64]. Several clinical trials for cell therapy are currently underway, such as the transplantation of dopaminergic neurons for Parkinson’s disease [65], retinal pigment epithelial cells for macular degeneration, and neural progenitor cells for SCI [66]. Trials to date have generally shown the safety of such transplanted neural cells with efficacy yet to be established. Similar to dopaminergic neurons, the differentiation of stem cells into SMNs has been investigated for many years, and multiple protocols have been developed to reliably generate SMNs [67,68]. Human PSCs can be generated from human embryos (embryonic stem cells) or engineered from adult tissue (induced pluripotent stem cells—IPSCs). IPSCs avoid the ethical controversy of sacrificing a human embryo and offer the potential to create an immune-compatible cell from the individual receiving this treatment. Currently, generating cells from each individual may be cost-prohibitive, but there are current efforts devoted to manipulating IPSCs to evade the immune system, which could allow for universal, readily available, cryo-banked cell lines [69,70]. Most protocols differentiate IPSCs by introducing small molecules that recapitulate the patterning pathways seen during development to generate a mixed population of neural cells with a ventral spinal cord identity, including SMNs, interneurons, and glia [68]. These cells may be transplanted as a mixed population or SMNs may be isolated in an additional step. An alternative approach is to directly engineer cell fate through forced expression of cell-specific factors, such as transcription factors or miRNA to generate a clonal, homogenous population of SMNs [71]. 

Proof of principle for this approach has been demonstrated in several rodent studies using a sciatic nerve transection model, where SMNs transplanted into the distal segment have survived, extended axons, and demonstrated functional innervation [72,73,74,75,76]. Despite support for this approach in rodent studies over several decades, there has been little progress in advancing this concept clinically. Only two reports have transplanted human cells into the immunosuppressed rodent. Both utilized a mixed population of IPSC-derived ventral cord neural cells. One report demonstrated survival, axon extension, NMJ formation, and prevention of muscle atrophy, but failed to show electrophysiological response to stimulation [77]. The other showed axon elongation to NMJs with a weak but reproduceable electrophysiological response [78]. Further investigation is necessary to establish reliable functional innervation of denervated muscle by transplanted human SMNs, including large animal models to demonstrate clinical feasibility of this approach on a scale closer to humans. 

### 3.3. Electrical Stimulation of Denervated Muscles

EMS is increasingly recognized as a promising approach for addressing the challenges associated with LMN injuries [79]. While a number of studies have been published indicating that this is not an effective method for recovering muscle health, these studies all fail to provide sufficient energy to drive a functional contraction in the denervated muscles [80,81]. Stimulation parameters for activation of denervated muscle is distinct from clinically available NMES. EMS requires higher intensity and longer stimulation pulse durations directly applied to the muscle fibers to achieve contraction [82,83]. EMS has had very limited application in the United States because the FDA limitation for energy provided by stimulators precludes the energy levels required for the activation of a denervated muscle. EMS can directly activate muscle fibers, bypassing the need for motor axons, and achieve a full contraction depending on the length of time of denervation. In fact, the amplitude of these contractions does diminish with time and based on the severity of the histological changes within the muscle (Figure 3) [84,85]. These induced contractions can then be applied as a training protocol. As is the case with innervated muscle, repetitively contracting denervated muscle will not only arrest muscle atrophy but also promote blood circulation within the affected area. With time this can reverse the histological changes associated with denervation [86,87]. 

In order to achieve these effects, EMS parameters need to be optimized for each individual, taking into account the severity of the injury, the muscles targeted, the chronicity of denervation, and the goals of therapy [88]. The electrical properties of muscle fibers change following denervation, including alterations in ion channel function and distribution, which contribute to an increasing threshold for activation. As more time passes, the caliber of muscle fibers and the proportion of fibers within a region of muscle falls. This will again compromise the robustness of the contraction achieved. All of these factors must be considered when initiating the training protocol.

Several animal models have demonstrated that short-term denervated muscle changes can be reversed with electrical stimulation [89,90]. Long-term denervated lower extremity muscles of paralyzed patients were also shown to be effectively restored in 25 individuals with complete cauda equina and conus medullaris injuries several years following denervation using this technique [91]. This study showed that 2 years of home-based EMS restored muscle fiber microstructure and mass [92,93]. Notably, in some participants, electrical stimulation of denervated muscles generated sufficient force to allow full bodyweight bearing suggesting that the use of EMS as an FES intervention could achieve standing and stepping. As a result of this study, a new technology was developed for human use with parameters that could transcutaneously activate long-term denervated muscles (Stimulator RISE, Schuhfried Medizintechnik GmbH, Vienna, Austria). 

Despite the success of clinical studies, controversy exists as to the benefit of EMS for nerve transfer interventions. That is, while the health of the muscle is clearly improved, it is unclear whether axons can now effectively reinnervate such a muscle in the chronic period following denervation when axons have not occupied these terminal nerve branches or neuromuscular junctions for such a long time. Early studies indicated varied outcomes on muscle reinnervation in the setting of such stimulation. Some indicated a negative effect [94], others no effect [80,81], and still others indicated a positive effect [95,96]. One of the negative effects proposed was a potential adverse impact of EMS on axonal sprouting during reinnervation [94]. However, several animal studies have demonstrated a beneficial effect, with improved morphology and functional capacity of the reinnervated muscles which had been stimulated compared with non-stimulated controls [97,98,99,100,101,102]. Improved autophagy flux within the SCs of the distal nerve segment has also been proposed as a possible mechanism for improved regeneration after EMS [103]. In human studies on facial paralysis, no evidence was found to indicate that electrical muscle stimulation hindered reinnervation or increased synkinesis [104,105].

In summary, recent studies suggest that EMS can mitigate denervation effects before reinnervation occurs by preserving muscle excitability and countering atrophy. However, it remains unclear as to whether late chronic muscle that has been recovered by this method can be reinnervated at that stage.

## 4. Conclusions

SCI rehabilitation has seen dramatic advances with the introduction of nerve transfers to restore arm and hand function. Still, this has only been available to patients who are either referred within the first year following their injury or who have a very limited LMN injury as a consequence of the type of spinal trauma suffered. Preserving these denervated muscles or, even better, reversing the effects of the denervation-related changes would open these surgical rehabilitation options to a far wider population. In addition, it would open the potential for implementing nerve transfer strategies to overcome the long regenerative distances required to recover lower extremity function. 

There are a number of promising modalities under development currently that may allow us to accomplish these goals. Sensory preservation has promise in preserving the distal nerve sheaths, but whether it can effectively delay the loss of muscle integrity is uncertain. Despite a promising single case report, there have been no follow-up studies in humans. Stem cell transplantation offers a simple intervention that could eliminate the need for an arduous training protocol and would potentially provide all of the factors required to maintain nerve and muscle until more appropriate axons can be provided. As previously described, there is strong support for this approach in rodent studies. However, there are only two reports that used human donor cells with mixed results. Moving forward will require rigorous pre-clinical studies investigating the capacity for transplanted human-specific SMNs to functionally innervate and maintain muscle viability. This also includes whether support cells (glia, interneurons) or neurotrophic factors are necessary, whether exogenous stimulation is needed to realize the full effect, and the quantity of cells required for a given muscle target. Electrical stimulation clearly can reverse denervation changes in muscle, but it requires an intensive training plan and it is not yet clear whether these recovered muscles can be effectively reinnervated. This is due to the fact that while there are powerful trophic effects on the muscle, there would be little corresponding effect on the nerve which likely continues to see progressive degeneration with time. Despite the promising findings in the RISE study in Europe, clinical investigation of EMS in the United States has been limited. Although EMS parameters were shown to be safe in the European studies targeting very large muscle groups, there may still be safety concerns for the increased energy necessary to stimulate these denervated muscles in the US as the FDA has not yet approved these energy levels for clinical care. Additional clinical studies will be critical to allay these fears and demonstrate the efficacy of EMS as a powerful tool for both reviving and maintaining muscle viability. A combination of these modalities may be more powerful than any in isolation. For example, coupling sensory preservation to occupy endoneurial tubes with axons while rehabilitating the muscle with stimulation may work together to address all of the targets required to keep the target available for reinnervation.

We believe large animal studies are critical for translational efforts in nerve regeneration once proof of principle is established in rodents. The rodent has a remarkable capacity for native regeneration and repair. In addition, distances to muscle targets must be longer than can be modeled in rodents in order to relate to the problems encountered in the human. This has been recognized and porcine models have recently been proposed as a large animal [106]. Addressing neuromuscular degeneration following denervation would be transformative in the field of peripheral nerve repair and would expand both nerve transfer and FES strategies to a broader set of applications and larger proportion of the SCI population. 

## Abbreviations

BCI	Brain–computer interface
CNS	Central nervous system
EMS	High-energy muscle stimulation
FAP	Fibro-adipogenic progenitor
FES	Functional electrical stimulation
IPSC	Induced pluripotent stem cell
LMN	Lower motor neuron
NMES	Neuromuscular electrical stimulation
NMJ	Neuromuscular junction
PNS	Peripheral nervous system
SC	Schwann cell
SCI	Spinal cord injury
SMN	Spinal motor neuron
UMN	Upper motor neuron
